# K_Ca_-Related Neurological Disorders: Phenotypic Spectrum and Therapeutic Indications

**DOI:** 10.2174/1570159X21666221208091805

**Published:** 2023-05-18

**Authors:** Aqeela Zahra, Ru Liu, Wenzhe Han, Hui Meng, Qun Wang, YunFu Wang, Susan L. Campbell, Jianping Wu

**Affiliations:** 1 School of Chemistry, Chemical Engineering and Life Sciences, Wuhan University of Technology, Wuhan 430070, China;; 2 Department of Zoology, University of Sialkot, Sialkot 51310, Pakistan;; 3 Beijing Tiantan Hospital, Capital Medical University, Beijing 100070, China;; 4 Advanced Innovation Center for Human Brain Protection, Capital Medical University, Beijing 100070, China;; 5 National Clinical Research Center for Neurological Diseases, Beijing 100070, China;; 6 Taihe Hospital, Hubei University of Medicine, Shiyan 442000, China;; 7 Animal and Poultry Sciences, Virginia Polytechnic Institute and State University, Blacksburg, VA, United States

**Keywords:** Potassium channels, channelopathies, modulators, pharmacology, epilepsy, action potential

## Abstract

Although potassium channelopathies have been linked to a wide range of neurological conditions, the underlying pathogenic mechanism is not always clear, and a systematic summary of clinical manifestation is absent. Several neurological disorders have been associated with alterations of calcium-activated potassium channels (K_Ca_ channels), such as loss- or gain-of-function mutations, post-transcriptional modification, *etc*. Here, we outlined the current understanding of the molecular and cellular properties of three subtypes of K_Ca_ channels, including big conductance K_Ca_ channels (BK), small conductance K_Ca_ channels (SK), and the intermediate conductance K_Ca_ channels (IK). Next, we comprehensively reviewed the loss- or gain-of-function mutations of each K_Ca_ channel and described the corresponding mutation sites in specific diseases to broaden the phenotypic-genotypic spectrum of K_Ca_-related neurological disorders. Moreover, we reviewed the current pharmaceutical strategies targeting K_Ca_ channels in K_Ca_-related neurological disorders to provide new directions for drug discovery in anti-seizure medication.

## 1 INTRODUCTION

Ion channels play a critical role in membrane transport activities that are essential for the optimal physiological function of the body. It has been suggested that pathogenic changes in ion channel activity and transporter function might cause diseases, such as migraines with hemiplegic symptoms, epilepsy, and cardiac arrhythmias, which are commonly referred to as ‘ion channelopathies’ [1]. Among ion channels, potassium channels (K^+^ channels) may control neuronal excitability during development due to their variable gating capabilities and vast temporal and spatial expression patterns [2]. For example, they may modulate the resting membrane potential, control the repolarization rate of action potentials (AP), and spike frequency adaptation patterns [3]. Consequently, K^+^ channel dysfunction is associated with a variety of neurological disorders, including epilepsy (Ohthara syndrome, Temple-Baraitser syndrome, and malignant migrating partial seizures of infancy), arrhythmia, myokymia, and developmental abnormalities of neural crest-derived tissues (Andersen syndrome) [4]. However, the underlying molecular mechanisms and their correlation with corresponding clinical manifestations are unclear.

Recent studies have found that even very little alterations in the activity of calcium-activated potassium channels (K_Ca_) may have significant effects on neuronal growth and cognitive ability [5]. The K_Ca_ channel is a large family of potassium channels found in almost every human cell type, including nerve cells, muscles, secretory cells, *etc*. K_Ca_ channels are formed with three different isoforms, which are classified by their activation and conductance features, namely big conductance K_Ca_ channels (BK channels), small conductance K_Ca_ channels (SK channels), and intermediate conductance K_Ca_ channels (IK channels). However, more studies are focusing on the BK and SK channels with little knowledge about IK channels so far [6]. Increases in cytosolic Ca^2+^ *via* voltage-gated Ca^2+^ channels and intracellular sources (endoplasmic reticulum, mitochondria) can gate all types of K_Ca_ channels and subsequently terminate the AP. They typically function by connecting membrane potential to intracellular Ca^2+^ concentration, which is crucial in a variety of physiological processes, such as regulating the interval of APs, presynaptic neurotransmitter release, and postsynaptic cell firing in certain neurons [7]. K_Ca_ channels can also be triggered by other messengers like kinases, protein phosphatases, G proteins, *etc*. [8]. In addition to the direct electrophysiological role, K^+^ channels have been reported to participate in diverse physiological processes *via* regulating cellular signaling pathways, including gene expression, and proliferation

In this review, we focused on the molecular and cellular properties of K_Ca_ channels. We comprehensively reviewed the loss- or gain-of-function of each K_Ca_ channel and attempted to bridge the phenotypic-genotypic spectrum in K_Ca_-related neurological disorders. Here, we systematically summarized the association between all the subtypes of K_Ca_ channels and K_Ca_-related neurological channelopathies. Moreover, we reviewed the current pharmaceutical strategies targeting K_Ca_ channels in K_Ca_-related neurological disorders to provide new directions for drug discovery in anti-seizure medication.

## 2 BK CHANNELS IN K_Ca_-RELATED NEUROLOGICAL DISORDERS

### 2.1 The Physiological Properties of the BK Channel

The BK channel (also known as Slo1 or K_Ca_1.1) was first discovered in the chromaffin cell membrane [9]. To date, documentation of BK channel expression shows that it is highly expressed in a variety of mammalian cells and tissues, including neurons, excretory cells, cardiac and smooth muscles, as well as skeletal and internal sensory hair cells of the ear [10, 11] (Table **1**). In the brain, BK channels are widely expressed in numerous regions, including the cerebral cortex and hippocampus [10].

**Fig. (1):**
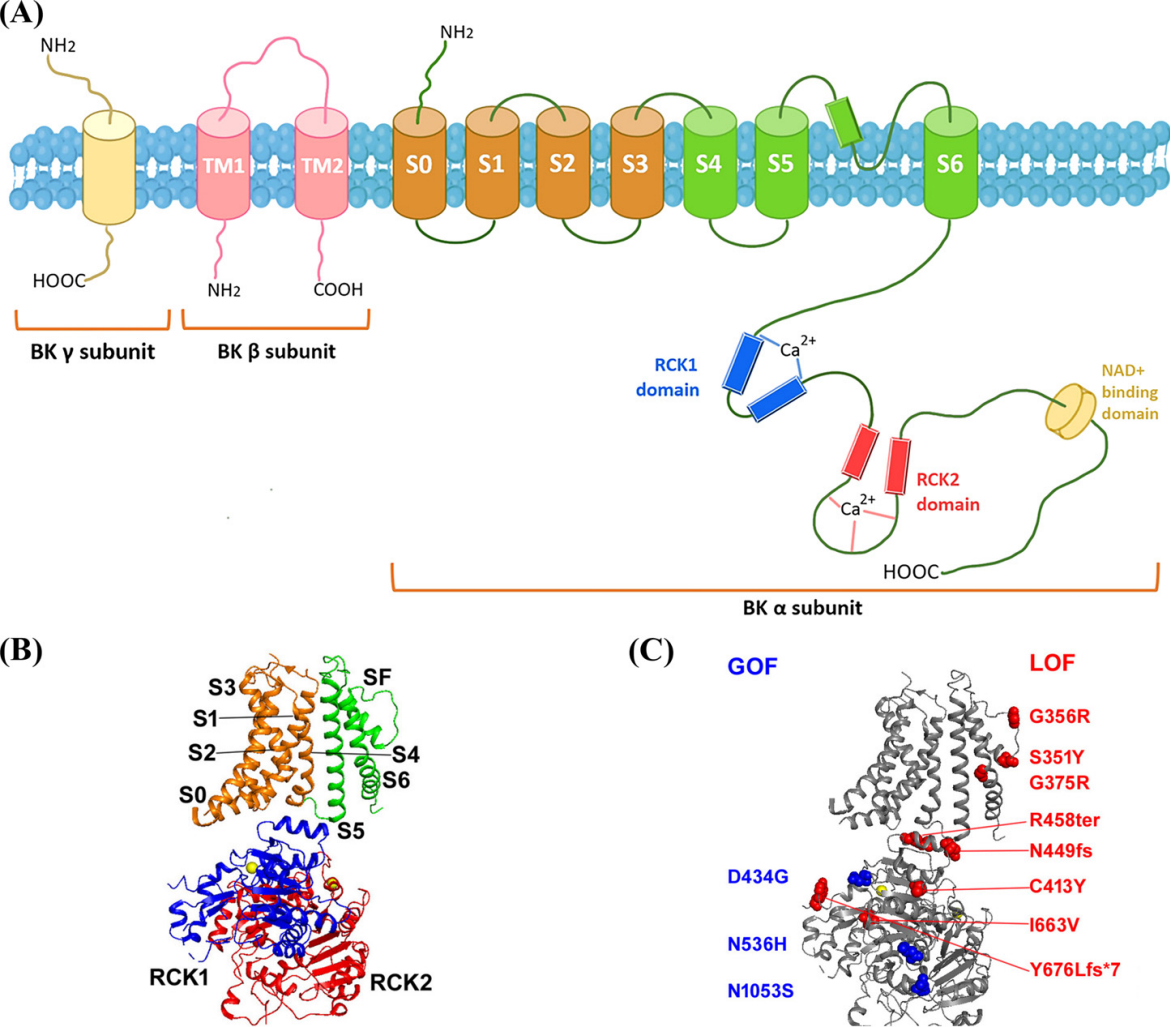
A topography of the BK channel with reported human mutation sites. (**A**) Each α-subunit of the BK channel has seven transmembrane domains (S0~S6), with the pore area between S5 and S6. A functional channel is made up of four of these subunits, with the C-terminus being one of the longest of all potassium channels. The C-terminus comprises two regulators of potassium conductance (RCK) domains, RCK1, and RCK2, which stack on top of one another to create a gating ring beneath the channel opening pore. (**B**) A single BK channel cry-EM subunit (PDB entry: 6V38). S0-S6 denote the transmembrane segments; Selectivity filter (SF) denotes the selectivity property; the two Ca^2+^ molecules that are coupled to the channel are represented in yellow. (**C**) GOF mutation sites are shown in blue, LOF mutation sites are in red mapped onto the BK channel structure and Ca^2+^ ions bound to the channel are shown in yellow circles. (**B** and **C** Copyright from 2022 [Jianmin Cui]. All Rights Reserved) [15].

BK channel is a tetrameric structure composed of a pore-forming domain (BK-α) encoded by *KCNMA1* and two accessory domains (BK-β and BK-γ). The BK-α subunit of the BK channel in mammals shares 98 percent of its amino acid sequence with that of other members of the voltage-activated and ligand-gated K^+^ channel superfamily, which participates in voltage-dependent electrophysiological property of the BK channel like voltage-dependent (K_v_) channels and other types of potassium channels. There are seven transmembrane domains in BK-α (S0~S6) and a substantial cytoplasmic C-terminus (Table **1**, Fig. **1**). The extracellular N-terminus and presence of the S0 transmembrane region distinguish BK from other voltage-gated potassium channels and allow for the interaction with additional proteins β and γ subunits [12]. There are two K^+^ conductance regulator (RCK) domains in the intracellular C-terminus of the BK channel, the allosteric gating mediated by RCK1 and RCK2, containing 2 unique high-affinity Ca^2+^ binding sites. BK channel activation is influenced by electrostatic interaction between Mg^2+^ and RCK1. Furthermore, RCK1 controls the sensitivity of channels to Zn^2+^ and Cd^2+^ [13-15].

BK channels are widely believed to prevent neural high-frequency and repeated firing. Reduced Cereblon (Crbn), a well-known target of the immunomodulatory drug thalidomide, was originally identified to cause human intellectual disability. It has previously been shown that BK channel activity is modulated by crbn in primary cultured neurons [16]. To better understand the biological underpinnings of intellectual impairment, research has shown that synaptic abnormalities are the most common factor in cognitive dysfunction. It was discovered that behavioral defects in Crbn KO mice could be restored by treatment with the BK blocker paxilline [17]. This strongly suggests that Crbn KO mice exhibit abnormally increased BK channel activity-induced decreased excitatory presynaptic release, which is closely related to cognitive dysfunction. However, BK channel activity paradoxically increases the firing of early high-frequency spikes of rat hippocampal pyramidal cells [18]. Other studies reported that increasing BK channel activity tends to promote synchronization of neuronal activity in animal models, such as Angelman syndrome. Sun *et al.* found that enhanced BK channel activity led to increased intrinsic excitability in hippocampal neurons and contributed to subsequent synchronization in the network [19]. BK channels are now receiving more consideration as potential therapeutic targets because of their wide distribution and involvement in an extensive range of physiological processes [20].

### 2.2 Physiological Modulation of the BK Channels

When the BK channel is activated by increasing intracellular Ca^2+^ concentration (Ca^2+^) as a result of membrane excitability and depolarization, it terminates the action potential with K^+^ outflow, resulting in hyperpolarization. This characteristic is advantageous in excitable cells because it enables negative feedback control of Ca^2+^ influx [21, 22]. In addition to Ca^2+^, the cytosolic Mg^2+^ and H^+^ can also modulate BK channels. The H^+^ activates the BK channel *via* electrostatic interactions between histidine residues and a nearby negatively charged residue. A homologous motif in the RCK1 domain regulates both the stimulatory and inhibitory activities of H^+^ and Ca^2+^, allowing bidirectional linkage of cell metabolism and membrane electrical excitability. Moreover, it is possible that the affinity of BK channels towards H^+^, which is associated with cytoplasmic neural acidification, is an important element in the cessation of epilepsy events [23]. In addition to being regulated by several ions, enzyme-mediated modifications, such as ubiquitination, palmitoylation, and myristoylation, also influence BK channel expression and activity. Furthermore, endogenous modulators, including arachidonic acid, nitric oxide, zinc, GMP, cGMP, and cAMP-mediated phosphorylation of the channel, may also control BK channel function (Table **1**).

Studies also revealed that cysteine string protein α (CSPα) regulates BK channel expression [24]. CSPα is a synaptic vesicle-associated protein that is broadly expressed in the nervous system and displays unique anti-neurodegenerative properties [25]. The CSPα is identified as a major regulator of BK channel density in the neuronal plasma membrane. CSP null mice expressed 2.5-fold more BK channels than wild-type mice. Additionally, BK channel levels were significantly enhanced in neuroblastoma cells generated from the murine central nervous system (CNS) after the expression of a dominant negative variant of CSPα containing a mutant HPD (residues 43-45) sequence. The HPD motif is required for the heterotrimeric CSPα chaperone

complex to function properly. Mutations of CSPα in the N terminal J domain or central cysteine string region led to an increase in total and cell surface BK channel expression, resulting in greater BK channel current density [26]. Loss of CSPα function alters BK channel expression in the intact (CNS), which may lead to altered neuronal membrane excitability and contribute to the pathogenesis of neurodegeneration associated with either genetic loss or dysfunction of CSPα [27].

### 2.3 BK Channel-Linked Neurological Disorders

Both GOF and LOF mutations in *KCNMA1* have been associated with neurological diseases, resulting in a spectrum of moderate to severe abnormalities. A summary of GOF, LOF of *KCNMA1,* as well as overlap symptoms, particularly in the areas of neurodegenerative disorders, is shown in Table **2**. Previous studies have found that LOF mutations in *KCNMA1* might result in widespread hyperexcitability, causing seizure activity and other related neurological conditions [28]. In addition, GOF mutations in *KCNMA1* are linked to numerous neurological conditions, suggesting that alterations in the activity of BK channels may affect the brain's equilibrium. BK channels are found in both inhibitory and excitatory neuronal networks, and their influence on firing varies depending on their expression [10, 29-36].

As mentioned before, the BK channel is a tetramer composed of four pore-forming subunits. It seems that 2 GOF and 10 LOF variants of the BK are mainly located on the pore gate domain (PGD; S5, pore and S6 segments) and cytosolic domain, which regulate the function of BK *via* changing the gating characteristics and activating by intracellular Ca^2+^. Although there are fewer GOF variants of *KCNMA1*, 20/37 patients carried GOF variants while 13/37 carried LOF variants. However, reviewing 37 patients with KCNMA1-linked channelopathy, paroxysmal non-kinesigenic dyskinesia (PNKD) was reported in 17 of 20 GOF patients and only 2 of 13 LOF patients. In addition, although seizure is a predominant symptom and equally associated with both GOF and LOF *KCNMA1* mutations, all nine GOF *KCNMA1* mutations patients with seizures experienced absence seizures. Notably, patients with both GOF and LOF *KCNMA1* showed developmental delay and intellectual disability; a subset of neurodevelopmental symptoms has only been described in patients with LOF *KCNMA1*. Due to the lack of detailed clinical description, it is difficult to determine if particular symptoms are specific to GOF *versus* LOF channel mutations [37].

BK channel accessory subunit-β family, which is composed of four different isoforms and encoded by *KCNMB1-4*, is linked to a variety of nervous system diseases. For instance, alcohol escalation was accelerated, and the chronic tolerance to ethanol-induced sedation and hypothermia was reduced in β1 knockout mice [38, 39]. As for the β2 subunit, it is encoded by *KCNMB2* and also linked to hippocampal sclerosis, a concomitant neuropathological characteristic of Alzheimer's disease [40]. On the other hand, *KCNMB3* deletion increases the risk of idiopathic generalized epilepsy in children. However, the precise mechanism of how such mutation affects neuronal due to the low levels of the β3 subunit is still unclear [40,41]. The β3 encoding gene *KCNMB3* is duplicated in some patients with the dup (3q) syndrome [43]. Furthermore, the β4 subunit, which is highly expressed in specific brain regions like the lateral hypothalamus, the Purkinje layer, and the striatum, has been found to control ethanol tolerance at the molecular, cellular, and behavioral levels [44]. In another study, after hyperpolarization (AHP), latency decreased and the firing rate elevated in dentate gyrus granule cells in β4 knockout mice. Moreover, nonconvulsive strokes occurred spontaneously in β4 knockout mice [45].

Single nucleotide polymorphisms (SNPs) are an important class of genetic mutations. When an SNP disrupts secondary structural elements (for example, replacement of proline in the alpha helix region), the resulting mutation often has an impact on the entire protein structure and function. Missense SNPs in *KCNMA1* have been related to human neurological diseases. An SNP in *KCNMB4* is also associated with an increased risk of developing mesial temporal lobe epilepsy [46]. Many studies revealed a possible link between Alzheimer’s disorder (AD) pathophysiology (onset age or duration of symptoms) and an SNP in the gene *KCNMA1*, rs16934131 [40, 47]. In addition, numerous cases of mental disability have been associated with a potential LOF SNP mutation in *KCNMA1* [48, 49]. Studies on the Fragile X Mental Retardation Protein (FMRP), which regulates dendrite-specific protein synthesis and is essential for brain development, have demonstrated the relationship between the BK channel and cognitive disorders. FMRP deficiency results in excessive AP broadening during repetitive activity, and increased presynaptic Ca^2+^ influx, in cortical pyramidal neurons [50]. However, these presynaptic actions are mediated selectively by BK channels *via* the interaction of FMRP with the BK channel’s regulatory β4 subunits [51].

### 2.4 Binding Site of BK Channel Compounds

There are two putative high affinity Ca^2+^ binding sites in the BK channel domains: one at Asp362/Asp367 in the RCK1 domain (these residue numbers are derived from the mbr5 sequence of the mouse subunit) [52] and the other in a region known as the Ca^2+^ bowl, which has a number of Asp residues in it [53], situated inside the RCK2 domain [54]. By binding with certain residues in the cytosolic domain, additional signaling chemicals, like carbon monoxide and heme [55, 56], may also change the gating characteristics of the channel. The activation of ion channels by intracellular ligands is often described as a conformational change in the cytosolic domain caused by ligand binding, which then pulls to open the activation gate at the peptide link between the membrane-spanning and cytosolic domains, called the tugging model [57, 58]. In contrast, Mg^2+^ activates BK channels by pushing the voltage sensor through electrostatic interaction and emphasizes the connection among side chains in distinct structural domains, also known as a nudging model. Since the Ca^2+^ sensitivity of BK channels is dependent on the length of the C-linker [59], it has been hypothesized that the activation of the channel by Ca^2+^ takes place through a tugging model. However, new evidence suggests that Ca^2+^ binding to the two separate high-affinity sites activates the channel with different mechanisms, and that the processes underlying Ca^2+^ dependent activation through the two sites may be more complicated.

The distinction between the Ca^2+^ binding sites was first described in a study where the Ca^2+^ bowl was mutated [60]. The results showed that the channel retained a partial sensitivity to Ca^2+^ while sustaining its sensitivity to Cd^2+^. It has been hypothesized that BK channels have a second Ca^2+^ binding site that may also bind to Cd^2+^ to activate the channel. Later research confirmed that the Cd^2+^ sensitivity of RCK1 is due to its putative second Ca^2+^ binding site (Asp362/Asp367, where Asp362 has a small influence on Ca^2+^ sensitivity) [61]. A recent study discovered that mutating ten residues in the N-terminal area of the RCK1 domain from the AC region may specifically modify the Ca^2+^-dependent activation originating from the location in RCK1 [62].

Furthermore, the change of voltage and Ca^2+^-dependent activation by the disease-associating mutations also provides unique insights for further understanding BK molecular mechanisms. Since G375R [32] is located in the diglycine hinge for the BK channel activation gate [63], it is possible that this mutation interacts with the opening of the activation gate and that G356R [32] eliminates the selectivity filter [64]. The effects of cytosolic domain mutations on voltage-dependent activation or intrinsic gate opening in BK channels are unknown. Some examples of such mutations are N1053S [48], N536H [65], C413Y, and I663V. Genetic data from human patients, biophysical characterizations, and animal models of GOF or LOF BK channel mutations suggest that BK channels might be a suitable therapeutic target for treating neurological diseases.

### 2.5 The BK Channel-pharmacology

For the majority of genetic channelopathies, the only option for pharmacological therapy is to modulate the particular activity of mutant ion channels. Precision medicine for *KCNMA1* and other channelopathies begins with classifying patient mutations into functional classifications. In addition, GOF, LOF, and possibly benign mutations in the BK channel activity are linked with overlapping symptoms. It is unclear whether specific BK channel agonists or antagonists would truly result in the intended consequence on neuronal activity. It is difficult to determine which BK channel components target which neuron or muscle loci in *KCNMA1*-linked channelopathy without more precise information. Research on BK channel pharmacotherapy has been in progress for over two decades; these efforts aim primarily to address neurological dysfunction caused by LOF mutations in BK channel activity [66].

BK agonists have been found in a variety of substances, including those that are endogenous, naturally occurring, and synthetic [67]. In the endogenous class are heme and heme-breakdown products [68], long-chain free polyunsaturated acids, cytochrome P450 metabolites, epoxygenase, and lipoxygenase [69], as well as 17-estradiol [70]. The naturally existing class includes several compounds found in herbs, roots, and leaves that have been utilized in traditional medicine to treat asthma and other conditions caused by myocyte dysfunction, such as dehydrosoyasaponin I (DHS-I) [70, 71]. Ca^2+^ channel inhibitors, like dihydropyridines (DHPs), are another possible FDA-approved medication for normalizing BK channel activity associated with GOF mutations. Some cases of dyskinesia can be treated with DHPs, nifedipine and nimodipine, as well as the non-DHP verapamil that is now being utilized to treat refractory epilepsy and hyperkinetic movement disorders [73]. Furthermore, the study on BK channels has been accelerated significantly by the development of specific inhibitors, such as iberiotoxin, paxilline, lolitrem B, and penitremA. Mehranfard *et al.* indicated that paxillin and iberiotoxin reversed firing characteristics of dentate gyrus granule cells in the chronic phase of pilocarpine-induced status epilepticus, suggesting that BK channels may have the potential to treat epilepsy [74].

The synthetic class includes GoSlo-SR, which is a newer family of BK channel activators that include NS004 and NS1619, as well as the more powerful and selective NS11021 [75]. When injected two hours after the onset of occlusion in rat models of persistent large-vessel stroke, Bristol-Myers Squibb (BMS-204352), a fluoro-oxindole potassium channel opener, demonstrated significant cortical neuroprotection [76]. Similarly, NS11021 activated the BK channel in a concentration-dependent manner at concentrations greater than 0.3 M by parallel-shifting the channel activation curves to more negative potentials. The single-channel analysis demonstrated that NS11021 improved channel open probability without influencing single-channel conductance by modifying gating kinetics [77]. Furthermore, NS11021 was shown to be a highly selective and efficient BK channel activator, making it an ideal candidate for investigating the physiological and pathological aspects of BK channels.

## 3 SK CHANNELS IN K_Ca_-RELATED NEUROLOGICAL DISORDERS

### 3.1 The Physiological Properties of the SK Channel

**Fig. (2):**
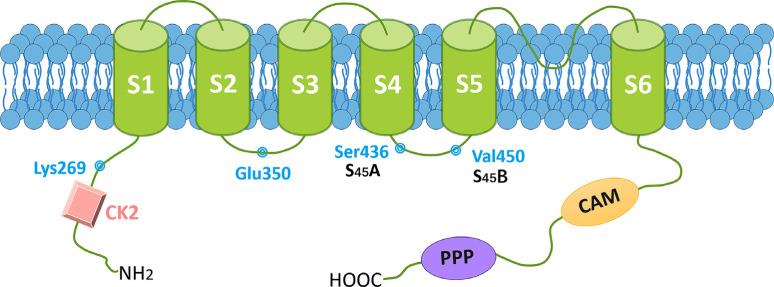
A topography of the SK channel with reported human mutation sites. SK channel has six transmembrane domains (S1~S6), with the pore region between S5 and S6. Both the N-terminus and the C-terminus point toward the cytoplasm. The CaM binding site, protein kinase CK2 and protein phosphatase (PPP) binding sites are indicated by yellow, pink, and purple, respectively. GOF mutation sites are shown in blue.

Three distinct SK channels encoded by *KCNN1-3* exist in the mammalian brain: SK1 (K_Ca_2.1, *KCNN1*), SK2 (K_Ca_2.2, *KCNN2*), and SK3 (K_Ca_2.3, *KCNN3*). These channels are structurally similar throughout their transmembrane cores (80-90%) but different in sequence at their N and C termini (Fig. **2**). Two SK2 isoforms have been identified: SK2-long (SK2-L), having a 207 amino acid N-terminal extension and SK2-short (SK2-S) [78]. SK channels are abundant throughout the nervous system and significantly expressed in certain brain areas, including the hippocampus, amygdala [79], and thalamus [80]. In addition, brain areas, such as the cortex, hippocampus, and limbic system, all have low levels of SK3 channels [81]. In many cases, both SK1 and SK2 channels are co-located in the same neurons, which may explain why heteromeric SK1/SK2 channels are preferentially assembled [82]. SK channels have vital functions outside the nervous system, such as regulating blood pressure and metabolic processes, as shown in Table **1**.

SK channels are required for the proper function of all excitable cells. SK currents are activated when calcium enters neurons *via* voltage-gated calcium channels, which are activated during the AHP following an AP. As soon as SK channels open, K^+^ is expelled from the cell, resulting in higher negative membrane potential, which regulates neuronal excitability and spike firing rates [83]. They may also be functionally coupled to post-synaptic calcium sources, such as N-methyl-d-aspartate (NMDA) and nicotinic acetylcholine receptors, calcium released from intracellular ryanodine or inositol 1,4,5-trisphosphate receptors [82].

SK channels modulate membrane excitability in CA1 neurons and regulate hippocampal neural plasticity [84, 85], as well as play a significant role in learning and memory due to their expression in the post-synaptic membrane of glutamatergic terminals, where they regulate neurotransmission and induce neural plasticity [66]. Many studies revealed that learning is impaired by increased activity in SK channels [86, 87]; in contrast, animal studies showed that SK channel antagonists improve learning and memory [88].

### 3.2 Modulation of the SK Channels

SK channels have positive and negative gating modulators that change the Ca^2+^ response curve of these Ca^2+^/calmodulin-gated channels to the left or right, respectively, increasing or decreasing the channel's sensitivity to Ca^2+^ [25]. Negatively modulated molecules are more likely to cross biological barriers if they are uncharged at physiological pH. In contrast, the more recently disclosed (-)CM-TMPF and the structurally similar (-)B-TMPF operate as SK1-selective positive and negative gating modulators having EC_50_ or IC_50_ values of 24 and 31 nM, respectively [89]. Dichloro-EBIO and the more potent benzimidazolone 1-EBIO are the most often utilized positive gating modulators [90]. They serve as important *ex vivo* chemicals used in brain slices, endothelia and epithelia, and smooth muscle preparations.

Additionally, in one study, melatonin induced downregulation of *KCNN1* and 2 expressions restored cognitive impairment caused by cerebral hypoperfusion [91]. Ethanol-induced synaptic excitability in the ventral but not the dorsal hippocampus has recently been linked to changes in the expression of the SK2 and GluA2 subunits in the synaptosomal membrane of the hippocampal neurons [92]. Morphine sensitization may potentially be influenced by increased SK2 channel-mediated negative feedback of NMDA receptor [93]. Withdrawal of cocaine eliminates the neuroplasticity mediated by SK2 in the nucleus accumbens neurons [94]. These findings suggest that drug-induced plasticity alters the activity of SK channels, which may play a crucial role in rewarding behavior.

### 3.3 SK Channel-Linked Neurological Disorders

It is not surprising that aberrant levels and/or activities of SK channels have been associated with a variety of CNS disorders as these channels play key roles in synaptic transmission, rhythmic activity, and other CNS processes. Lee *et al.* indicated that the three amino acid replacements in SK3 channels were found in patients with Zimmermann-Laband syndrome (ZLS), a rare genetic disorder characterized by abnormalities of the head and facial (craniofacial) area and the hands and feet [95]. The *KCNN3* mutation has recently been also implicated in bipolar disorder [96]. Overexpressing SK3 channels in mice showed impairments in long-term potentiation and memory deficits as well as decreased high cognitive function in the hippocampus [96-98].

Decreased SK3-mediated current and increased neuronal excitability in the nucleus accumbens core are essential processes that enhance the desire for alcohol during abstinence [99]. Similarly, the expression and function of SK channels were dramatically decreased in the pilocarpine model of epilepsy [100]. Decreased SK activity was also shown to be related to increased seizure activity in Dravet syndrome [101]. Schizophrenia patients also have a spontaneous mutation in the *KCNN3* gene [102]. Based on these findings, balanced SK channel activity is required for proper neurodevelopment and cognitive functions (Fig. **2**). Unfortunately, due to the lack of detailed clinical description and reported variants, it is limited to determine if particular symptoms are specific to GOF *versus* LOF channel mutations and find ‘hot spots in the channel.

### 3.4 The SK Channels-Pharmacology

SK channel is inhibited by bee venom toxin apamin by interacting with an outer pore histidine residue which is expressed in all SK subtypes *via* an allosteric mechanism [103, 104]. The SK channel blockers like Syllatoxin (isolated from the scorpion Leiurus quinquestriatus) or leiurotoxin I, a stronger scorpion toxin, have similar efficacy to apamin [105]. An entirely new benzimidazole derivative, NS8593, has just been described as an inhibitor of the K_Ca_ channel with a completely different chemical structure. A gating modulator, NS8593, shifts the Ca^2+^ activation curve of K_Ca_ channels approximately 10-fold to the right, decreasing their Ca^2+^ sensitivity. NS8593 subsequently inhibits cloned SK1, SK2, and SK3 with IC50 values of 420, 600, and 730 nM at a concentration of 500 nM [106]. UCL1684 and UCL1848, which are less utilized blockers, are equally strong as apamin in inhibiting the SK channel. Previous research has shown that bicuculine methiodide has an apamin-like effect on dopamine neurons that enhances the effects of NMDA receptor activation.

The development of specific SK channel activators resulted in the discovery of NS309, one of the most effective pan-SK2 activators and a critical mechanical tool compound [107]. NS309 and SKA-31 equally stimulate all three SK channels [108]. CyPPA and its recently reported derivative NS13001 are examples of subtype-specific SK activators [109]. The notion of utilizing SK activators is based on the discovery that SK channel silencing in deep cerebellar neurons causes ataxia in mice [110]. In contrast, the treatment of mice with spinocerebellar ataxia type-2 with NS13001 alleviated movement disorders and delayed neurodegeneration in Purkinje cells [111]. Riluzole, a more effective neuroprotectant, causes SK channels to shift to the left and increases AHP in cultured hippocampal neurons by increasing their Ca^2+^ sensitivity [112-118] (Table **3**).

## 4 IK CHANNELS IN K_Ca_-RELATED NEUROLOGICAL DISORDERS

### 4.1 The Physiological Properties of the IK Channel

The intermediate-conductance channel (IK) encoded by *KCNN4* was initially identified in erythrocytes by Gardos in 1958 [119] and is known as SK4 because it is ∼40% identical to the three SK channels [25]. It was finally cloned from pancreatic, placental, or T-lymphocytes in 1997 [26-28]. Additionally, IK was also discovered in the lung, salivary glands, distal colon, and prostate; and was absent in the heart, brain, liver, kidney, and skeletal muscle [120-123]. The expression of the IK channel in some enteric neurons has been shown [124]. IK and BK channels are structurally nearly identical. Each IK channel has six transmembrane domains (numbered S1-S6) and has an active pore, which is located between S5 and S6 [125]. Acidic residues in the IK channel -subunit S4 transmembrane region render it insensitive to voltage changes compared to the BK channels. Since the S0 transmembrane domain is absent in IK channels, the enhanced Ca^2+^ sensitivity of these channels is due to the calmodulin-binding domain on the α-subunit C-terminal region of the protein that binds Ca^2+^.

The IK channel is generally activated by Ca^2+^ release from intracellular stores or Ca^2+^ inflow through store-operated channels (SOCs) in the endoplasmic reticulum (ER). In addition, activation of the IK channel in excitable cells causes hyperpolarization, which, in turn, enhances the Ca^2+^ driving force for SOC entrance. This enormous influx specifically activates voltage-dependent ion channels, and the subsequent K^+^ and Ca^2+^ outflow of ions hyperpolarizes the membrane and enhances the driving force for Ca^2+^ efflux [126]. Recent studies suggest that IK may be expressed in neurons as well and have a role in the lowering of AHP [127]. However, the significance of the IK channel in neurons remains unclear.

### 4.2 IK Channel-linked Neurological Disorders and Pharmacology

In studying how Aß oligomers trigger reactive astrogliosis, researchers have discovered that IK channels can increase the driving force for Ca^2+^ influx, making them a potential therapeutic target for AD. Additionally, APP/PSEN1 AD mice with the deletion of the *KCNN4* mutation in the hippocampus were shown to improve memory impairments and neuronal loss [128]. Despite the lack of clear evidence that IK channels are involved in the pathogenesis of AD, multiple investigations have shown that oxidative stress impairs IK function in older animals [129].

Peptide blockers of the IK channels include charybdotoxin, (ChTx), a 37-amino acid scorpion toxin, which binds to the outer vestibule through two salt bridges while inserting its core lysine residue into the specific segment [130]. ChTx also inhibits BK channels, making it an extremely promiscuous blocker of channels [131]. Additionally, maurotoxin, a 34-residue scorpion toxin cross-linked with four disulfide bridges, is the most powerful peptide blocker of IK [132]. More surprisingly, maurotoxin tends to block Kv voltage-gated channels with higher IC_50_ (100 pM). ShK (IC_50_ 30 nM), BgK (IC_50_ 172 nM), and margatoxin (IC_50_ 450 nM) are several other toxins that inhibit IK at high concentration [133]. The triaryl-methane IK channel blockers clotrimazole, TRAM-34, and senicapoc, interact with threonine 250 in the pore loop and valine 275 in S6, as demonstrated by the fact that mutations of these residues completely abolish the sensitivity of IK channel to triaryl-methanes [134]. Recently, it was shown that negative-gating modulation is also responsible for the actions of some Di-benzoates, such as RA2 [1,3-phenylenebis(methylene)bis(3-fluoro-4-hydroxybenzoate], which blocks both SK and IK channels with comparable efficacy [135]. Even though its binding site has to be fully discovered, the inner vestibule of both channels (IK and SK) is large enough to accommodate it. Sankaranarayanan *et al.* developed a series of benzothiazoles, including SKA-31 and SKA-121, that have increased selectivity for the IK channel and demonstrated that selective IK activation could decrease blood pressure in mice [108].

EBIO (ethylbenzimidazolone) was the first compound to be shown to activate IK currents in T84 cells when applied at high micromolar concentrations. Evidence suggests that EBIO enhanced chloride secretion by activating IK channels located on the cell surface of T84 monolayers and secretory endothelia [136]. EBIO activates human cloned IK channels (hIK) by raising the single-channel open probability in a calcium-dependent manner, leading to an apparent leftward shift of the Ca^2+^ activation curve by order of magnitude. Activation of the IK channel by 8-methoxypsoralen, the methylxanthines, caffeine and theophylline, and a clotrimazole-sensitive K^+^current in mouse jejunum preparations (100 mM to 1 mM) are further examples of chemical classes that have been found to activate the IK channel. The most potent therapeutic drugs for BK, SK, and IK channels are shown in Fig. (**3**).

## CONCLUSION

K_Ca_ channels modulated by intracellular Ca^2+^ concentration play a critical role in neuronal excitation. Growing studies have reported that mutations in K_Ca_ channels are associated with neurological disorders, such as developmental, epileptic encephalopathies, and severe psychomotor and intellectual disabilities. In this review, we first summarized the molecular and cellular properties of three K_Ca_ channels, BK, SK and IK channels. Next, we comprehensively reviewed the LOF and GOF mutations of each subtype of the K_Ca_ channel and discussed the corresponding disease to broaden the phenotypic-genotypic spectrum of K_Ca_-related neurological disorders. Moreover, we explored the various underlying modulatory mechanisms of K_Ca_ channels and the current pharmaceutical strategies to provide new directions for developing more potent and selective treatment strategies for K_Ca_-related neurological disorders.

## Figures and Tables

**Fig. (3):**
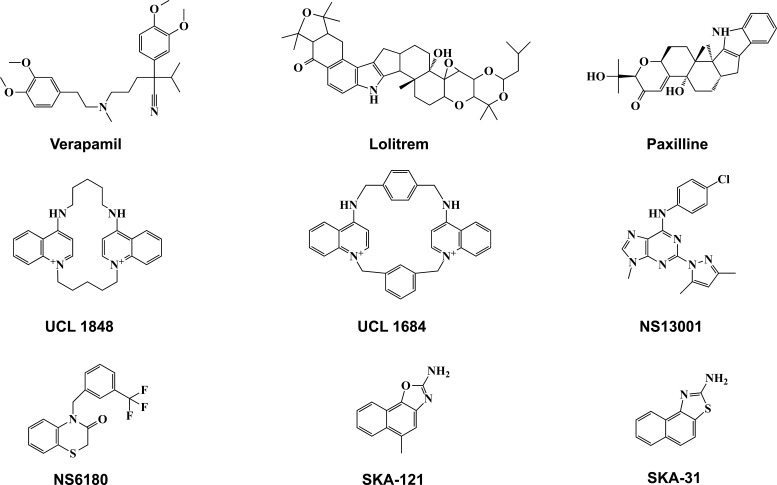
Names and structures of BK, SK, and IK channel modulators.

**Table 1:** A summary of the structural, physiological, and pharmacological properties of K_Ca_ channels.

**Properties**	**BK Channel**	**SK Channel**		**IK Channel (SK4)**
		**SK1 and SK2 Channels**	**SK3 Channel**	
Structure characters	β and γ subunits, seven transmembrane domains, 2 RCK domains in intracellular C terminal.	Six transmembrane domains with amino acid sequence (S-Y-A) between the S3-S4 domain.	Six transmembrane domains with amino acid sequence (S-Y-T) between the S3-S4 domain.	Six transmembrane domains with shorter N-terminus.
Channel gating	Voltage, Ca^2+^, Mg^2+^, H^+^, *etc*	Ca^2+^ *via* CaM		Ca^2+^ *via* CaM.
CNS expression	Olfactory bulb, cortex, basal ganglia, caudate putamen, CA1 region, cerebellum.	CA1 region, amygdala, thalamus, cerebellum.		Midbrain, brain cell nuclei.
Electrophy-siological function	Fast AHP in neurons, dendritic excitability, synaptic plasticity.	Medium AHP in neurons, dendritic excitability, synaptic plasticity.		Volume regulation in erythrocytes.
Biological function	Intercellular Ca^2+^↑	EDHF response.		Ca^2+^ signal in T-cell, Proliferation; EDHF response.
Pharmacology	Blockers: Dihydropyridines, Iberiotoxin, Paxillin, Penitrem A Openers: NS004, NS1619, BMS-204352	Blockers: Apamin, Leiurotoxin I, UCL1684, Openers: Dichloro-EBIO, NS309, riluzole.		Blockers: Charybdotoxin, Maurotoxin, SKA-31, Margatoxin, TRAM-34. Openers: EBIO, NS309, riluzole, methylxanthine.

**Abbreviations**: AHP, After hyperpolarization; CaM, calmodulin; EDHF, Endothelium-Derived Hyperpolarizing Factor; Dichloro-EBIO, Dichloro-Ethylbenzimidazolone.

**Table 2:** Mutations in *KCNMA1* associated with human neurological diseases.

**Clinical Phenotypes**	**Homo ** **Variants**	**Location**	**Mutant Types**	**Function Test; Model**	**Results**
**GOF**					
E, PNKD [30]	D434G	RCK domain	Missense mutation	Yes; transfected oocytes or subcloned CHO cell	BK channel opening increased due to a three- to five-fold increase in Ca^2+^ sensitivity.
E, DD [31]	N995S	NAD domain	Missense mutation	Yes; pcDNA3.1 cloned by human *KCNMA1* cDNA	BK channel opening increased with higher sensitivity to the voltage change.
**LOF**					
Ataxia, tremor, apraxia, hypertelorism [32]	S351Y	Pore domain	Missense mutation	Yes; NA	The BK channel is eliminated, and the K^+^ current is blocked.
Cognitive delay, axial hypotonia, ataxia dysarthria [32]	G356R	Pore domain	Missense mutation	Yes; NA	BK channel is eliminated, and the K^+^ current ranging from -160~60 mV is blocked.
DD, visceral and cardiac malformation dysplasia, dysmorphic features [32, 33]	G375R	S6 domain	Missense mutation	Yes; NA	The BK channel is eliminated, and the K^+^ current is blocked.
Congenital abnormalities, DD, ID, axial hypotonia, ataxia [33]	C413Y	AC region of RCK	Missense mutation	Yes; NA	The amplitude of BK current is reduced, and its activation curves shift toward positive potentials.
DD, ID, axial hypotonia, ataxia [32]	I663V	RCK domain	Missense mutation	Yes; NA	The BK channel is eliminated, and the K^+^ current is blocked.
DD, E, cerebellar and corticospinal tract atrophy [34]	Y676Lfs*7	RCK domain	Frameshift mutation	No; NA	The activity of the BK channel decreased.
Speech delay, DD, ID, apraxia [33]	P805L	RCK domain	Missense mutation	Yes; NA	The amplitude of BK current is reduced, and its activation curves shift toward positive potentials.
E, PNKD [32]	D984N	RCK domain	Missense mutation	Yes; oocytes injected with cRNA	The amplitude of BK current is reduced, and its activation curves shift toward positive potentials.
**Overlap GOF/LOF**					
PNKD, DD, visual impairment [33]	E884K	RCK domain	Missense mutation	No; NA	Not determined.
PNDK, DD, ID [35]	G354S	Pore domain	Missense mutation	Yes; transfected Xenopus oocytes	Reduced BK channel activity and delayed activation.
DD, ID, myoclonic seizures [36]	R458Ter*	AC domain	Nonsense mutation	No; NA	Putative truncations (premature truncation mutations).

**Abbreviations**: AC, Cytosolic domain; RCK, Regulators of K^+^ conductance GOF, gain of functional mutation; LOF, loss of functional mutation; E, epilepsy; PNKD, Paroxysmal Non-Kinesigenic Dyskinesia; DD, developmental delay; ID, intellectual disability, NA; Not available.

**Table 3:** GOF Mutations in *KCNN1-4* associated with human neurological diseases.

**Clinical Phenotypes**	**Gene Type**	**Homo ** **Variants**	**Location**	**Mutant Types**	**Function Test; Model**	**Results**
ZLS, DD, ID, hypotonia [113]	*KCNN1*	G350D	S2-S3 domain	Missense mutation	Yes; pcDNA3.1 cloned by human *KCNN3* cDNA	Increased Ca^2+^ sensitivity and faster channel activation in mutant SK channel.
DD, ID, ZLS [113]	*KCNN1*	S436K	S_45_A domain	Missense mutation	Yes; subcloned CHO cell	
ZLS, ID, DD, PDA [113]	*KCNN2*	K269E	N-terminus	Missense mutation	Yes; HEK293T cells	
DD, Hypo-tonia [114]	*KCNN3*	V555F	NA	Missense mutation	Yes; NA	
ID, DD [114]	*KCNN3*	V539del	NA	Nonsense mutation	Yes; NA	
DD, Mild seizures [114]	*KCNN3*	A287S	NA	Missense mutation	Yes; NA	
INCPH [115]	*KCNN3*	V450L	S_45_B domain	Missense mutation	Yes; NA	
HX [116]	*KCNN4*	R352H	CaMB domain	Missense mutation	Yes; HEK293T cells	Increased 10 folds Ca^2+^ sensitivity and current density in encoded mutant channel.
DHSt [117]	*KCNN4*	V282M	S6 domain	Missense mutation	Yes; CD34+ cells and K562 cells	Increased Ca^2+^ sensitivity in mutant channel.
DHSt [118]	*KCNN4*	V282E	S6 domain	Missense mutation	Yes; CD34+ cells and K562 cells	

**Abbreviations**: DD, developmental delay; GOF, gain of functional mutation; ID, intellectual disability; INCPH, idiopathic non-cirrhotic portal hypertension; NA; not available. PDA, patent ductus arteriosus; ZLS, Zimmermann–Laband syndrome. CaMB, Calmodulin domain; HX, Hereditary xerocytosis; DHSt, Dehydrated hereditary stomatocytosis.

## LIST OF ABBREVIATIONS

AC: Cytosolic Domain
AHP: After Hyperpolarization
AP: Action Potential
BK Channels: Big Conductance K_Ca_ Channels
CaM: Calmodulin
CNS: Central Nervous System
CSPα: Cysteine String Protein α
DD: Developmental Delay
DHPs: Dihydropyridines
E: Epilepsy
EBIO: Ethyl-benzimidazolone
EDHF: Endothelium-derived Hyperpolarizing Factor
GABA_A_: Gamma-aminobutyric Acid A Receptors
GOF: Gain of Function
ID: Intellectual Disability
IK Channels: Intermediate Conductance K_Ca_ Channels
K_Ca_: Calcium-activated Potassium Channel
LOF: Loss of Function
NMDAR: N-methyl-D-aspartate Receptor
PNKD: Paroxysmal Dyskinesia
RCK: Regulators of K^+^ Conductance
SK Channels: Small Conductance K_Ca_ Channels
